# The influence of lifestyle, menstrual function and oral contraceptive use on bone mass and size in female military cadets

**DOI:** 10.1186/1743-7075-4-17

**Published:** 2007-08-06

**Authors:** Jamie A Ruffing, Jeri W Nieves, Marsha Zion, Susan Tendy, Patricia Garrett, Robert Lindsay, Felicia Cosman

**Affiliations:** 1 Clinical Research and Regional Bone Centers, Helen Hayes Hospital, West Haverstraw, NY, USA; 2 Departments of Medicine and Epidemiology, College of Physicians and Surgeons of Columbia University, New York, USA; 3 United States Military Academy, West Point, NY, USA

## Abstract

**Purpose:**

To determine the influence of menstrual irregularity, oral contraceptive use and other factors on bone mineral density (BMD) and bone size at different skeletal sites in 135 college-aged fit women.

**Methods:**

Menstrual history, oral contraceptive use, exercise history, and nutritional factors including calcium, caffeine, and alcohol intake as well as tobacco use were determined by written survey. Height, weight and fitness levels were measured. Spine and hip BMD were measured by dual x-ray absorptiometry (DXA), calcaneus BMD by peripheral DXA, and tibial bone mineral content (BMC) and size by peripheral Quantitative Computed Tomography (*p*QCT).

**Results:**

The mean age was 18.4 ± 0.8 years. Weight and prior exercise were positively related to BMD at most skeletal sites and to tibial bone size. Milk intake was positively related to calcaneal BMD, tibial BMC and cortical thickness. Fracture history was an important predictor of spine, hip and heel BMD. Women who had ≥ 10 menstrual cycles in the year prior to BMD measurement had higher BMD at all sites as well as a greater tibial mineral content and cortical thickness than women who had oligomenorrhea/amenorrhea (≤ 9 cycles in the prior year; all p < 0.05). Oral Contraceptive (OC) users had significantly lower BMD in the spine (*p *< 0.02) and calcaneus (*p *= 0.04), smaller tibial periosteal circumference and lower tibial mineral content (*p *< 0.02) than non-OC users.

**Conclusion:**

In a population of fit, college-aged women, OC use and oligomenorrhea were associated with reduced BMD and bone size. Weight, as well as prior exercise and milk intake was positively related to bone density and size at some skeletal sites. Understanding these relationships would help improve skeletal health in young women.

## Background

Osteoporosis is a major public health concern as highlighted by the recent Surgeon General's report [1]. A key osteoporosis prevention strategy is to increase early accrual of bone mineral density (BMD). A higher BMD and larger bone size attained in childhood and maintained through the third decade of life has been related to a subsequent reduction in the risk of childhood fracture, stress fracture, osteoporosis and fractures related to osteoporosis later in life [2-5]. Therefore, it is important to understand factors that can be modified to improve the accrual of peak bone mass and increase in bone size in women [6-8].

Genetic factors account for 60–80% of the variance in peak bone mass [7,8]. Failure to achieve the genetically pre-determined complement of bone mass is often related to sub-optimal environmental and lifestyle conditions in women. Bone accrual can also be limited by eating disorders and oligo- and amenorrhea [9-11]. Oral contraceptive (OC) use may have an effect on bone accrual but its exact role is unclear. There is some evidence attributing a modest benefit of oral contraceptive use to spine and hip BMD. Alternatively, several recent studies have shown either no effect or negative effects of oral contraceptives on bone density. The impact of OC on bone size is also not well understood [12-15]. The type of contraception, age at first use and level of exercise may alter the impact of OC use on bone health [6,16,17]. Observational studies of OC use on bone mass may be confounded by the underlying reason for use as 4–9% of women use oral contraceptives for reasons other than birth control, including amenorrhea or oligomenorrhea [18]. There are many other factors that may positively influence BMD including high levels of physical activity and adequate calcium intake [6,19,20].

The purpose of this cross sectional study is to examine the influence of various factors associated with BMD, including calcium intake, exercise history, fitness level, body size, menstrual function and oral contraceptive use on bone mineral density and bone size in physically fit college age women.

## Methods

### Subjects

All cadets entering the United States Military Academy (USMA) Class of 2002, West Point, NY were sent a study information packet, including a sample consent form and study protocol, prior to their arrival at the Academy. The study objectives and the associated risks were described in a presentation during their first week at the Academy. Seventy percent of all eligible women in the class agreed to participate and provided written informed consent. There were no differences between those who elected to participate and those who did not with regard to race or age. One hundred and thirty five women consented to participate in a 4-year prospective study examining the determinants of peak bone mass and stress fractures. There are stringent medical requirements for entrance into USMA that led to the exclusion of women with severe polycystic ovarian disease and other diseases that affect BMD, including persons with uncontrolled thyroid disease, epilepsy, gastrointestinal disease or long term steroid use (Army Regulation 40–501, Chapter 2). Race was self-reported on USMA applications. The largest racial group was Caucasian (n = 107). Therefore analyses will focus on this group. However, racial differences in bone density will also be reported. The Institutional Review Board (IRB) of Keller Army Community Hospital (KACH), West Point, NY, approved the study.

### Lifestyle Assessments

Information on exercise, dietary and other lifestyle factors in the year prior to entering USMA was collected by questionnaire, allowing cadets to pick from categories to expedite data collection. Exercise was quantified as the number of exercise hours per week and divided into four categories of 1–3, 4–6, 7–10, or 11 + hours per week. Four questions assessed calcium intake including daily milk consumption (divided into 4 categories: none, <1, 1–2, or 3 or more 8 ounce glasses per day) and weekly servings of yogurt, cheese and high calcium content vegetables (0, 1–3, 4–6 or 7 + serving per week). From these responses, an average daily calcium intake was calculated (mg/day). Daily caffeine intake was divided into three categories: none, 1 to 3 and more than 3 caffeine containing drinks a day. Alcohol intake was categorized as: less than once a month, 1–3 times a month, 1 to 2 times a week, and 3 to 5 times a week. Tobacco use was assessed by type (dip, chew, or cigarettes), dose, and duration. Lifetime history of personal skeletal fracture and site of fracture were determined by questionnaire. Cadets reported their age at menarche, and current and prior use of birth control pills. Prior use was considered any use of oral contraceptives (OC) in the past for greater than 3 months. Current users included individuals taking OC for at least 3 months, including at entry to USMA. Frequency of menstrual cycles in the year prior to entering USMA was categorized as ≤9 cycles per year (amenorrhea/oligomenorrhea), or 10–12 cycles/year (normal) in the past year.

### Fitness and Anthropometric Measures

Academy personnel measured each cadet's height and weight at entry in standard athletic clothing in stocking feet and height was measured using a standard scale. Body Mass Index (BMI) was calculated as weight in kilograms divided by height in meters squared. Trained Academy personnel administered the Army Physical Fitness Test (APFT) to all cadets upon entry into USMA. This test measures upper body and abdominal strength and aerobic fitness. It includes the number of full body push-ups and bent leg sit-ups completed in two-minute segments as well as a timed two-mile run. These three events (sit-ups, push-ups and a 2 mile run) are then each converted into an age and gender standardized score ranging from 0–100 [21]. Faster running times, higher numbers of push ups and sit ups earn higher scores. The sum of these three scores represents the overall assessment of fitness with a range of 0–300.

### Bone Densitometry

The left total hip and lumbar spine (L1-L4) BMD (gm/cm^2^) were measured using a mobile DXA scanner (DPX-IQ, General Electric-Lunar, Madison WI). The coefficient of variation (%CV) *in vivo *was determined by measuring 8 individuals two times with repositioning. The %CV was 1.5% and 1.5% respectively for spine and left total hip in this population. During this study there was no impact of movement of the mobile DXA, based on no significant change in the phantom measurements as part of the daily quality control program for DXA measurements. BMD (g/cm^2^) of the left calcaneus was assessed using peripheral dual-energy x-ray absorptiometry (pDXA; Pixi, Lunar, Madison, WI). The coefficient of variation *in vivo *for the calcaneus pDXA was 1.0%. Peripheral QCT (Stratec XCT2000; Germany) was used to image a single slice at the distal tibia. To identify the site, the distal third of the tibia was determined by manual measurement of tibial length between the base of the patella and the styloid process (to the closest centimeter). The pQCT was positioned at the distal site after placing a positioning light of the gantry above the styloid process. The tibia was chosen because it is a major site of stress fractures in military cadets [8]. Bone mineral content (mg per 1 mm slice of bone), cortical thickness (mm), periosteal and endosteal circumferences (mm) were measured. Cortical bone was identified at a threshold above 710 mg/cm^3^. Cortical thickness was derived using the circular ring model, which calculates a mean cortical thickness from measures of total bone area and cortical bone area. The coefficient of variation was 2.2% for tibial mineral content and 3.2% for cortical thickness.

### Statistical Analysis

Student's t-tests were used to examine the differences in BMD among dichotomous lifestyle variables and non-parametric tests were used (Kruskal-Wallis Test) to test differences between variables that are not normally distributed such as menstrual cycle frequency. The relationships between lifestyle determinants and BMD were examined using Pearson's correlation analyses and, where appropriate, with linear regression to control for potential confounders. Effects of categorical lifestyle variables on BMD were also assessed using analysis of variance. The level of significance for alpha was set at 0.05 for these statistical tests. In Caucasian women, a multiple regression model was created using all covariates that had biologic plausibility and were significant in univariate analyses and variables with p < 0.1 were included in the model. All analyses were performed with SPSS statistical software (Version 13.0 for Windows, SPSS Inc., Chicago IL.)

## Results

### Population Characteristics

There were 135 women enrolled, including 107 Caucasians, 13 African Americans and 15 Asians. Table 1 provides a summary of the lifestyle variables from the baseline questionnaire for the Caucasian women. Overall, the women cadets in this study had healthy behaviours with 77% reporting they exercised more than 7 hours a week, and reporting an average daily calcium intake approximately equal to the recommended daily intake (RDI) for this age group (1000 mg calcium per day). Total APFT score was more than one standard deviation better than similar aged female recruits that enter basic training, a group that more closely represents the general population [21]. One component of the APFT, the run score equated to times for the two mile run test that ranged from 13 minutes 23 seconds to 22 minutes 39, with 50% of the women running faster than 8.37 pace per mile.

**Table 1:** Descriptive variables for Caucasian women (values are mean (standard deviation) unless otherwise stated)

Variable	All Women (n = 107)	Women with Normal Cycles (n = 81)	Women with Amenorrhea/Oligomenorrhea (n = 25)	Women on Oral Contraceptives (n = 12)	Women never on Oral Contraceptive (n = 95)
	Mean (SD)	Mean (SD)	Mean (SD)	Mean (SD)	Mean (SD)

Age	18.4 (± 0.8)	18.4 (± 0.8)	18.6 (± 0.9)	18.6 (± 1.0)	18.4 (± 0.7)
Age of Menarche (years)	12.8 (± 1.2)	12.6 (± 1.1)	13.6 (± 0.9) *	12.5 (± 1.2)	12.9 (± 1.1)
BMI (kg/mg^2^)	23.0 (± 2.3)	23.3 (± 2.4)	22.6 (± 2.0)	22.8 (± 1.8)	23.2 (± 2.4)
Height (inches)	65.7 (± 2.4)	65.6 (± 2.5)	66.1 (± 1.8)	64.6 (± 3.0)	65.8 (± 2.3)
Weight (pounds)	142 (± 17)	142.4 (± 17.8)	140.6 (± 15.3)	135.3 (± 12.8)	142.6 (± 17.4)
Army Physical Fitness Test (APFT 0–300)	207.8 (± 40.9)	206.0 (± 39.3)	215.1 (± 39.3)	188.3 (± 52.3)	211.8 (± 36.6)
Run score (range 0–100)	74 (± 22)	72.3 (± 22.1)	77.7 (± 22.0)	59.9 (± 25.7) **#**	76.1 (± 20.9)
Calcium Intake (mg/day)	1074 (± 463)	1167 (± 459)	1014 (± 389)	937 (± 419)	1142 (± 449)
Exercise (hrs/week) %, (n)					
1–3	3.0 (4)	2.9 (3)	3.4 (1)	0 (0)	1 (1)
4–6	19.5 (26)	19.4 (38)	20.7 (6)	41.7 (5)	15.6 (15)
7–10	33.8 (45)	36.9 (24)	24.1 (7)	33.3 (4)	38.5 (37)
11+	43.6 (58)	40.8 (42)	51.7 (15)	25.0 (3)	44.8 (43)
Milk (glass/day) %, (n)					
0	11.3 (15)	10.7 (11)	13.8 (15)	8.3 (1)	11.5 (11)
<1	29.3 (39)	23.3 (24)	48.3 (14)	25.0 (3)	25.0 (24)
1–2	46.6 (62)	52.4 (54)	27.6 (8)	50.0 (6)	49.0 (47)
3+	12.8 (17)	13.6 (14)	10.3 (3)	16.7 (2)	14.6 (14)
Caffeinated Drink/Day %, (n)					
None	24.1(32)	22.3 (23)	27.6 (8)	33.3 (4)	20.8 (20)
1–3	69.2 (92)	70.9 (73)	65.5 (19)	58.3 (7)	70.8 (68)
3+	6.8 (9)	6.8 (7)	6.9 (2)	8.3 (1)	8.3 (8)
Alcohol Consumption %, (n)					
Less than once a month	66.9 (91)	70.9 (73)	55.2 (16)	66.7 (8)	64.6 (62)
1–3 times a month	21.3 (28)	18.4 (19)	34.5 (10)	16.7 (2)	24.0 (23)
1–2 times a week	8.8 (12)	8.7 (9)	10.3 (3)	8.3 (1)	11.5 (11)
>3 times a week	1.5 (2)	1.9 (2)	0.0 (0)	8.3 (1)	0 (0)
Smokers %, (n)	3.0 (4)	2.9 (3)	3.4 (1)	8.3 (1)	2.2 (2)
History of Fracture (% yes); n	34.1 (46)	38.8 (40)	24.1 (7)	33.3 (4)	37.5 (36)

*p < 0.05 versus women with normal cycles

# p < 0.05 versus women never on oral contraceptives

Women on oral contraceptives had a significantly lower run score than women who never took oral contraceptives (p = 0.02). There were no other significant differences in population characteristics between women on oral contraceptives as compared to women who never used oral contraceptives (Table 1). Most women (97%) were tobacco free, and 10% admitted to consumption of more than two alcoholic beverages per week (although alcohol intake may be underestimated in this underage population). Thirty-four% of women reported previous fractures. Approximately 11% of the women currently used oral contraceptives and there was no reported use of Depo-Provera. All women had experienced menarche and the women with oligomenorrhea/amenorrhea reported reaching menarche a year later than those women with normal cycles in the past year (p < 0.05; Table 1). There were no other significant differences between women with normal cycles and women with oligomenorrhea/amenorrhea.

### Bone Density Measures

**Figure 1:**
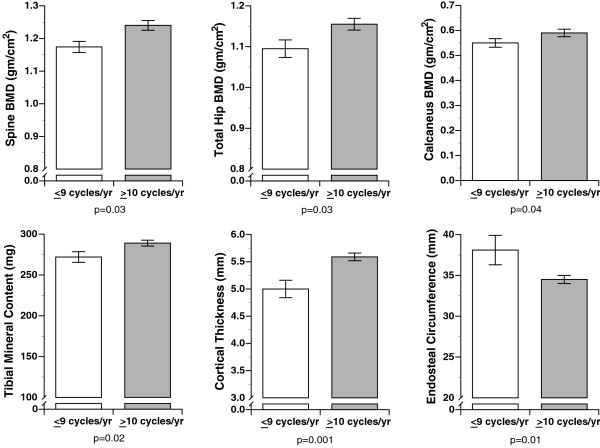
**The impact of menstrual function on bone mass and size**. Menstrual function was defined as normal (≥ 10 cycles/year) versus oligomenorrhea/amenorrhea (≤ 9 cycles/year). Bone mineral density was measured at the spine, hip and heel; and at the tibia, bone mineral content, cortical thickness and endosteal circumference were determined. All values are mean ± standard errors.

**Figure 2:**
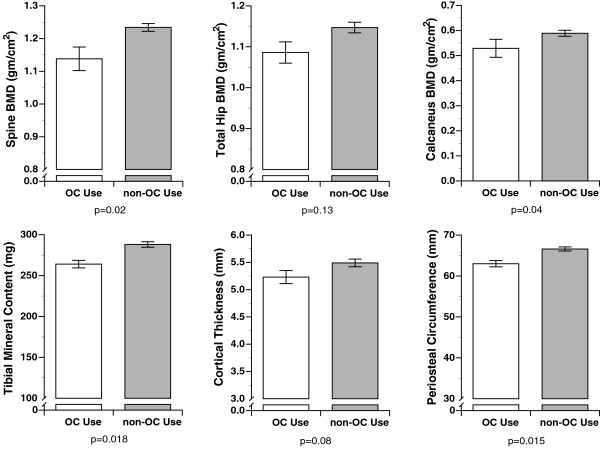
**The impact of oral contraceptive use on bone mass and size**. Oral contraceptive use was defined as OC use for at least three months versus never users. Bone mineral density was measured in these two groups at the spine, hip and heel; at the tibia, bone mineral content, cortical thickness and periosteal circumference were measured. All values are mean ± standard errors.

T-scores for the entire population were calculated based on the reference population from Lunar for the spine (average T-score = +0.6) and for the total hip (average T-score = +1.2) using the NHANES database. BMD results for the calcaneus, left total hip, spine (L1-L4) and tibia as well as tibial dimensions are reported by menstrual function category (Figure 1) and oral contraceptive use (Figure 2) and discussed below.

### Racial Differences

Although there were no significant racial differences in total hip BMD, African American women had slightly higher spine and calcaneus BMD when compared to Caucasians (*p *< 0.05). However, all races had significantly higher calcaneal, spine and hip BMD than the population average (*p *< 0.025). African American women had significantly larger periosteal circumference and tibial mineral content when compared to the other races (*p *< 0.02). However, there were no racial differences seen in cortical thickness.

### Predictors of BMD in Caucasian Women

#### Lumbar Spine and Total Hip

Milk consumption and total calcium intake had no effect on BMD at the spine or hip. There was no effect from caffeine, alcohol or smoking on spine or hip BMD. BMD was slightly higher (3%) in the spine and hip (4%) when comparing the highest exercise level to the other levels for the year prior to entering USMA (hip, p = 0.06; spine, p = 0.14). APFT and run score did not correlate significantly with spine or total hip BMD. Women with prior fractures had higher BMD at both the spine (1.26 ± 0.14 vs. 1.20 ± 0.10) and total hip (1.18 ± 0.12 vs. 1.12 ± 0.12) compared to those who did not have a prior fracture (both p < 0.04).

Age at menarche was not a determinant of spine or hip BMD. Women who had either amenorrhea or oligomenorrhea in the prior year had significantly lower spine and hip BMD (Figure 1; both p = 0.03). Never-users of OCs had significantly higher BMD in the spine when compared with current OC users (*p *= 0.02; Figure 2). Similar but not significant differences were found at the hip (p = 0.13; Figure 2).

BMI, height and weight were all modestly correlated with both spine BMD (r = 0.23, r = 0.36 and 0.41, all p < 0.01) and total hip BMD (r = 0.31; r = 0.21, and 0.37; all p < 0.01). Each ten-pound increase in weight was responsible for an approximate 2.5% increase in BMD.

In a linear regression analysis, approximately 30% of the variation in BMD at the spine was explained by weight, cycles per year, current OC use and prior exercise (Table 2). At the total hip 28% of the variation in BMD was explained by weight, fracture history, number of menstrual cycles and prior exercise (Table 2).

**Table 2:** Multiple linear regression models for bone density in Caucasian females

Calcaneal BMD	0.118	+0.002(weight)	+0.021(milk intake)	+0.049(current OC use)	
(SE)	0.080	0.001	0.009	0.025	
*p*	0.141	0.001	0.024	0.052	
*R*^2^* = 0.275*					
Spine BMD	0.461	+0.003(weight)	+0.035(cycles/year)	+0.062(current OC use)	+0.025(exercise)
(SE)	0.125	0.001	0.013	0.036	0.014
*p*	0.001	0.001	0.011	0.087	0.078
*R*^2^* = 0.313*					
Total Hip BMD	0.617	+0.003(weight)	-0.049(fracture hx)	+0.035(cycle/year)	+0.032 (exercise)
(SE)	0.001	0.001	0.022	0.013	0.014
*p*	0.001	0.001	0.030	0.010	0.021
*R*^2^* = 0.286*					

#### Calcaneus

Total daily calcium intake was modestly correlated with calcaneal BMD (*r *= 0.194; p < 0.05). Women with the lowest daily milk intake (<1 glass per day), compared to all higher intakes of milk, had 13% lower calcaneal BMD (*p *< 0.01). Caffeine, alcohol, and tobacco usage were not significantly related to calcaneal BMD in the young women in our study population. Women with a personal history of fractures had, on average, 6.7% higher calcaneal BMD than those women with no personal fracture history (*p *< 0.03). Prior exercise was significantly correlated with calcaneal BMD (r = 0.37; p < 0.001).

Age of menarche was not related to calcaneal BMD. Women who reported either amenorrhea or oligomenorrhea had lower calcaneal BMD (Figure 1: *p *= 0.04). A comparison of current OC users to women without OC use showed lower calcaneal BMD in current OC users (Figure 2; *p *= 0.04).

BMI and weight were moderately correlated with calcaneal BMD (*r *= 0.414 and *r *= 0.450, both *p *< 0.01). Neither height nor fitness parameters were correlated with calcaneal BMD.

Calcaneal BMD in our population of Caucasian women was best predicted by body weight, glasses of milk consumed per day and current OC use. These variables accounted for 27.5% of the variation in calcaneal BMD (Table 2). Each additional 10 pounds of body weight was associated with a 0.02 g/cm^2 ^increase in calcaneal BMD.

#### Tibial Mineral Content and Geometry

Women with low milk intake had 6% lower tibial mineral content than those drinking 3 or more glasses of milk daily (*p *= 0.06). There was a marginal difference in cortical thickness at different milk intakes (*p *< 0.08) but there was no effect of milk on periosteal circumference. Total daily calcium intake had no apparent effect on tibial mineral content or geometry in our population. Caffeine intake had a borderline significant negative impact on tibial mineral content (*p *= 0.07), but no relationship with cortical thickness or periosteal circumference. There was no relationship between smoking or alcohol with tibial BMC, cortical thickness or periosteal circumference. There was no association seen between prior fracture and any tibial parameter.

Women reporting the lowest exercise category had 8% lower tibial cortical thickness and 6% lower tibial mineral content than women reporting the highest exercise levels. Additionally, when comparing the two highest groups with the two lowest exercise groups there was a significant difference in both cortical thickness (*p *> 0.01) and tibial mineral content (p > 0.04) but not periosteal circumference.

There was no significant relationship between age of menarche and any of the tibial parameters. Those women with oligomenorrhea or amenorrhea in the year prior to entering USMA had significantly smaller cortical thickness (*p *= 0.001) in part related to a larger endosteal circumference (*p *= 0.01), and these women also had significantly lower tibial BMC (*p *< 0.02; Figure 1). As compared to OC users, tibial BMC was higher and periosteal circumference was larger in non-OC users (both *p *= 0.02; Figure 2). There was slightly larger cortical thickness in non-OC users as compared to OC users (p = 0.08; Figure 2).

Height was positively correlated with tibial BMC (r = 0.39; *p *> 0.01), and periosteal circumference (r = 0.34; *p *> 0.01), but not with cortical thickness. Weight was positively correlated with the three tibial parameters: tibial BMC (r = 0.58; *p *> 0.01), cortical thickness (r = 0.25; *p *> 0.01), and periosteal circumference (r = 0.50; *p *> 0.01). BMI was moderately correlated with tibial mineral content (r = 0.43; *p *> 0.01), cortical thickness (*r *= 0.27; *p *< 0.01) and periosteal circumference (*r *= 0.43; *p *> 0.01). Of the fitness measures, run score was modestly correlated with both tibial mineral content and cortical thickness (r = 0.23, r = 0.24, respectively, both *p *< 0.04) but not periosteal circumference. APFT was not correlated with any tibial parameter.

Results of the regression analyses for each of the tibial parameters in Caucasian women are presented in Table 3. Regular menstrual function or OC usage and weight were important predictors in all models, while run score was important for only tibial mineral content and cortical thickness.

**Table 3:** Multiple linear regression table for Caucasian female tibial parameters

Mineral Content	54.836	+10.63(cycles/year)	+1.063(weight)	+0.395 (run)	+6.157 (exercise)
(SE)	(27.03)	(2.87)	(0.15)	(0.12)	(3.39)
*p*	(0.095)	(0.001)	(0.001)	(0.002)	(0.073)
*R*^2^* = 0.493*					
Cortical Thickness	2.141	+0.355(cycles/year)	+0.011 (run score)	+0.009 (weight)	
(SE)	(0.696)	(0.077)	(0.003)	(0.004)	
*p*	(0.003)	(0.001)	(0.00)	(0.034)	
*R*^2 ^= *0.296*					
Periosteal Circumference	42.42	+0.135 (weight)	+2.496 (OC use)		
(SE)	(4.42)	(0.027)	(1.377)		
*p*	(0.01)	(0.001)	(0.07)		
*R*^2 ^= *0.272*					

## Discussion

In this cohort of 107 young Caucasian women, we found that weight was an important and consistent positive determinant of bone mass at all skeletal sites and of tibial cortical thickness and periosteal circumference. Prior exercise levels were positively related to calcaneal BMD, tibial BMC and cortical thickness, and in multivariable models predicted tibial mineral content and spine and hip BMD. A component of physical fitness, run score, predicted tibial BMC and cortical thickness. Milk consumption had an important influence on bone density of the heel and was marginally related to tibial BMC and cortical thickness. Prior studies have suggested that milk-based calcium intake is an important factor in increasing bone density and bone size [19,20]. The use of oral contraceptives had a negative effect on calcaneal and spine BMD, tibial mineral content and bone size as evidenced by a smaller periosteal circumference at the tibia. We found that one of the strongest negative predictors of bone density was oligomenorrhea that occurred in 13% of women or amenorrhea that occurred in 5% of this population. This prevalence of amenorrhea/oligomenorrhea is slightly higher than what has been reported in a Danish study [22], although the Danish population was likely to have had lower levels of exercise than these women entering the USMA. In fact, women in USMA who had oligomenorrhea or amenorrhea (32% of whom were avid exercisers with >11 hours exercise/week) had lower bone mineral density at all skeletal sites (spine, hip and heel), less BMC at the tibia and a smaller tibial cortical thickness through an increase in endosteal circumference.

The fact that body weight was an important factor for skeletal health is not surprising since genetic factors, including weight, can account for between 60–80% of the variation in BMD [2,8,23,24]. In this extremely fit population, weight may have also been related to muscle mass. In previous studies, high muscle mass has been associated with higher BMD while low muscle mass was associated with poor low BMD[25].

The benefits of exercise on bone health are supported by this study. Both a measure of average weekly exercise in the prior year and run score, a fitness measure used by the Army, were found to be important predictors of bone density at the spine and hip, tibial mineral content and cortical thickness. Long bone cross-sectional growth is also strongly driven by mechanical load associated with increased weight and exercise during growth [26] and in this cohort weight was a significant predictor of periosteal circumference and exercise and weight influenced cortical thickness of the tibia.

The high prevalence of prior fracture experienced by the women in this study (34%) may be related more to an active lifestyle and selection of activities with higher loads or greater risk than other women in this age group, rather than to a difference in bone mass. In fact, a personal history of fractures was significantly related to higher BMD at three sites: spine, total hip and calcaneus. There was no relationship between fractures in these women and parental fractures. There was no difference in any of the fitness measures or amount of prior exercise between those with prior fractures and those without. The type of exercise, rather than the amount, might have been more important to personal fracture risk. Alternatively, this could be explained by the suggestion that individuals with a genetic predisposition to poor bone quality who engage in weight bearing exercise, gain more skeletal benefit from exercise than those without this predisposition [27].

Prior studies evaluating the impact of OC use on BMD at different skeletal sites has provided mixed results with no clear consensus[12-14,28]. There is some indication that OC use at younger ages may suppress endogenous production of hormones and that the OC replacement dose of estrogen is inadequate and may compromise the large increases in bone mass during peak bone mass accrual [16]. In this study of young women, 10.7% reported being current OC users. OC use was associated with significantly lower bone mineral density at the spine and heel, and slightly lower BMD at the hip (p = 0.13), in agreement with several prior studies. Furthermore, we found that OC users had less tibial BMC and a unique finding of smaller tibial size (perisoteal circumference). The OC users in this study did not report higher prior exercise levels or perform better on the running test. In this study, it was not known why women were prescribed OCs; it may have been because of menstrual irregularity or for contraception. If OCs were used for oligomenorrhea or amenorrhea, the residual deleterious effects of this could have created the BMD deficit. However, we did not exclude women with oligomenorrhea or amenorrhea from the non-OC users group, which if anything would bias the results toward no effect of OC on bone.

A unique aspect of our study was the ability to assess periosteal circumference and cortical thickness of the tibia as important measures of bone size and strength. Menstrual dysfunction was related to a smaller cortical thickness, in part, a result of a larger endosteal circumference. Conversely, the use of oral contraceptives led to a smaller periosteal circumference and this smaller bone size could result in less bone strength. A prior study of women aged 18–31 years on oral contraceptives revealed a reduced cross-sectional area and cross-sectional moment of inertia in the femoral neck, also indicating reduced mechanical strength [12]. Thus, as shown elsewhere, an interruption in normal menstrual function or any alteration in estrogenic state, such as oral contraceptive use may alter the mechanostat set point of bone [7]. Periosteal growth, which enlarges bone diameter, accelerates at puberty in males. However, in females, periosteal growth is inhibited by estrogen at puberty [28] and of interest we saw that females on OCs had a smaller periosteal circumference. Cortical thickness can also be increased by apposition of endocortical bone but in females with oligomenorrhea/amenorrhea this may be prevented, and may in part explain the significantly larger endosteal circumference we found in women who have abnormal menstrual function.

This study had some limitations. Our questionnaire included only a brief self-administered food frequency questionnaire. There were many dietary factors not assessed on our questionnaire that may be important determinants of bone health including total caloric intake, but due to time constraints our dietary assessment had limited scope. Milk consumption was on average very high in this cohort, limiting ability to see an effect, except when comparing highest to lowest intakes. Our population of women was very fit, with above average BMD, therefore our external validity is limited. Finally, the inherent problems associated with cross sectional studies including the establishment of a temporal relationship exist in this study.

## Conclusion

In conclusion, increased levels of prior exercise and better running score, a measure of fitness, are positive determinants of BMD at the spine and hip as well as tibial mineral content and cortical thickness in our population of physically active young women. Higher milk intake may also have a positive impact on skeletal health at the heel and tibia. Physicians, coaches and trainers should consider menstrual status when examining the health of their athletes, as it appears that oligomenorrhea or amenorrhea may be important to bone health, having a detrimental affect on both bone mass and cortical thickness. In addition, OC use for menstrual irregularity may not be a solution to menstrual irregularity since there may in fact be further detrimental impact on bone mass at various skeletal sites and bone size as assessed by periosteal circumference of the tibia. Attaining a high peak bone mass and ideal skeletal geometry is important to prevent both stress fractures and fractures related to osteoporosis later in life in women. Therefore, longitudinal studies are warranted to examine the impact of nutrition, exercise, menstrual function and OC use on accrual of peak BMD as well as peak bone size.

## Competing interests

The author(s) declare that they have no competing interests.

## Authors' contributions

JA Ruffing contributed to study design, data collection, data analysis and manuscript preparation. JW Nieves was co PI contributed to study design, data collection and manuscript preparation. M Zion contributed to data collection, data analysis and manuscript preparation. S Tendy contributed to data collection, site supervision, and manuscript preparation. P Garrett contributed to data collection and data analysis. R Lindsay contributed to study design and manuscript preparation. F Cosman was co PI contributed to study design, data collection and manuscript preparation. All authors have read and approved the final manuscript.

## References

[B1] (2004). Bone Health and Osteoporosis: A Report of the Surgeon General.

[B2] Medicine I (1998). Reducing stress fracture in physically active military women.

[B3] Goulding A, Jones IE, Taylor RW, Manning PJ, Williams SM (2000). More broken bones: a 4-year double cohort study of young girls with and without distal forearm fractures. J Bone Miner Res.

[B4] Melton LJ, Crowson CS, O'Fallon WM, Wahner HW, Riggs BL (2003). Relative contributions of bone density, bone turnover, and clinical risk factors to long-term fracture prediction. J Bone Miner Res.

[B5] Szulc P, Beck TJ, Marchand F, Delmas PD (2005). Low skeletal muscle mass is associated with poor structural parameters of bone and impaired balance in elderly men--the MINOS study. J Bone Miner Res.

[B6] Bass S, Pearce G, Bradney M, Hendrich E, Delmas PD, Harding A, Seeman E (1998). Exercise before puberty may confer residual benefits in bone density in adulthood: studies in active prepubertal and retired female gymnasts. J Bone Miner Res.

[B7] McGuigan FE, Murray L, Gallagher A, Davey-Smith G, Neville CE, Van't Hof R, Boreham C, Ralston SH (2002). Genetic and environmental determinants of peak bone mass in young men and women. J Bone Miner Res.

[B8] Rubin LA, Hawker GA, Peltekova VD, Fielding LJ, Ridout R, Cole DE (1999). Determinants of peak bone mass: clinical and genetic analyses in a young female Canadian cohort. J Bone Miner Res.

[B9] Cobb KL, Bachrach LK, Greendale G, Marcus R, Neer RM, Nieves J, Sowers MF, Brown BW, Gopalakrishnan G, Luetters C, Tanner HK, Ward B, Kelsey JL (2003). Disordered eating, menstrual irregularity, and bone mineral density in female runners. Med Sci Sports Exerc.

[B10] Drinkwater BL, Nilson K, Chesnut CH, Bremner WJ, Shainholtz S, Southworth MB (1984). Bone mineral content of amenorrheic and eumenorrheic athletes. N Engl J Med.

[B11] Soyka LA, Misra M, Frenchman A, Miller KK, Grinspoon S, Schoenfeld DA, Klibanski A (2002). Abnormal bone mineral accrual in adolescent girls with anorexia nervosa. J Clin Endocrinol Metab.

[B12] Burr DB, Yoshikawa T, Teegarden D, Lyle R, McCabe G, McCabe LD, Weaver CM (2000). Exercise and oral contraceptive use suppress the normal age-related increase in bone mass and strength of the femoral neck in women 18-31 years of age. Bone.

[B13] Kleerekoper M, Brienza RS, Schultz LR, Johnson CC (1991). Oral contraceptive use may protect against low bone mass. Henry Ford Hospital Osteoporosis Cooperative Research Group. Arch Intern Med.

[B14] Lloyd T, Taylor DS, Lin HM, Matthews AE, Eggli DF, Legro RS (2000). Oral contraceptive use by teenage women does not affect peak bone mass: a longitudinal study. Fertil Steril.

[B15] Weaver CM, Teegarden D, Lyle RM, McCabe GP, McCabe LD, Proulx W, Kern M, Sedlock D, Anderson DD, Hillberry BM, Peacock M, Johnston CC (2001). Impact of exercise on bone health and contraindication of oral contraceptive use in young women. Med Sci Sports Exerc.

[B16] Cromer BA (2003). Bone mineral density in adolescent and young adult women on injectable or oral contraception. Curr Opin Obstet Gynecol.

[B17] Hartard M, Kleinmond C, Kirchbichler A, Jeschke D, Wiseman M, Weissenbacher ER, Felsenberg D, Erben RG (2004). Age at first oral contraceptive use as a major determinant of vertebral bone mass in female endurance athletes. Bone.

[B18] Wegienka G, Baird DD (2003). Potential bias due to excluding oral contraceptive users when estimating menstrual cycle characteristics. Am J Epidemiol.

[B19] Cadogan J, Eastell R, Jones N, Barker ME (1997). Milk intake and bone mineral acquisition in adolescent girls: randomised, controlled intervention trial. Bmj.

[B20] Chan GM, Hoffman K, McMurry M (1995). Effects of dairy products on bone and body composition in pubertal girls. J Pediatr.

[B21] Headquarters DA (1998). FM 21-20: Physical Fitness Training.

[B22] Munster K, Helm P, Schmidt L (1992). Secondary amenorrhoea: prevalence and medical contact--a cross-sectional study from a Danish county. Br J Obstet Gynaecol.

[B23] Magnusson PK, Rasmussen F (2002). Familial resemblance of body mass index and familial risk of high and low body mass index. A study of young men in Sweden. Int J Obes Relat Metab Disord.

[B24] Martin RB (2002). Size, structure and gender: lessons about fracture risk. J Musculoskelet Neuronal Interact.

[B25] van Langendonck L, Claessens AL, Lysens R, Koninckx PR, Beunen G (2004). Association between bone, body composition and strength in premenarcheal girls and postmenopausal women. Ann Hum Biol.

[B26] van der Meulen MC, Ashford MW, Kiratli BJ, Bachrach LK, Carter DR (1996). Determinants of femoral geometry and structure during adolescent growth. J Orthop Res.

[B27] Suuriniemi M, Mahonen A, Kovanen V, Alen M, Lyytikainen A, Wang Q, Kroger H, Cheng S (2004). Association between exercise and pubertal BMD is modulated by estrogen receptor alpha genotype. J Bone Miner Res.

[B28] Seeman E (2003). Periosteal bone formation--a neglected determinant of bone strength. N Engl J Med.

